# Experiences of giving birth during the COVID-19 pandemic: a qualitative analysis of social media comments through the lens of birth integrity

**DOI:** 10.1186/s12884-022-05326-2

**Published:** 2023-01-16

**Authors:** Céline Miani, Antonia Leiße, Lisa Wandschneider, Stephanie Batram-Zantvoort

**Affiliations:** 1 Department of Epidemiology and International Public Health, School of Public Health, Bielefeld University, Universitätstr. 15, 33615 Bielefeld, Germany; 2 Sexual and Reproductive Health and Rights Research Unit, Ined, France

**Keywords:** Social media, Qualitative research, Respectful maternity care, COVID-19, Birth, Birth integrity

## Abstract

**Background:**

Social media offer women a space to discuss birth-related fears and experiences. This is particularly the case during the COVID-19 pandemic when measures to contain the spread of the virus and high rates of infection have had an impact on the delivery of care, potentially restricting women’s rights and increasing the risk of experiencing different forms of mistreatment or violence. Through the lens of birth integrity, we focused on the experiences of women giving birth in Germany as shared on social media, and on what may have sheltered or violated their integrity during birth.

**Methods:**

Using thematic analysis, we identified key themes in 127 comments and associated reactions (i.e. “likes”, emojis) posted on a Facebook public page in response to the dissemination of a research survey on maternity care in the first year of the COVID-19 pandemic.

**Results:**

Women contributing to the dataset gave birth during March and December 2020. They were most negatively affected by own mask-wearing –especially during the active phase of labour, not being allowed a birth companion of choice, lack of supportive care, and exclusion of their partner from the hospital. Those topics generated the most reactions, revealing compassion from other women and mixed feelings about health measures, from acceptation to anger. Many women explicitly formulated how inhumane or disrespectful the care was. While some women felt restricted by the tight visiting rules, those were seen as positive by others, who benefited from the relative quiet of maternity wards and opportunities for postpartum healing and bonding.

**Conclusion:**

Exceptional pandemic circumstances have introduced new parameters in maternity care, some of which appear acceptable, necessary, or beneficial to women, and some of which can be considered violations of birth integrity. Our research calls for the investigation of the long-term impact of those violations and the reassessment of the optimal conditions of the delivery of respectful maternity during the pandemic and beyond.

## Background

Over the past decade, there has been growing evidence that women^1^ worldwide experience sub-standard care during facility-based childbirth, ranging from non-adherence to evidence-based practices, non-consented care, neglect, disrespect, restrictions of autonomy, or unsatisfactory health facility conditions [1]. In 2014, the World Health Organization (WHO) called for global research, advocacy, and action to prevent and eliminate mistreatment during facility-based childbirth and has contributed to advance this quest ever since [2]. Based upon the latest evidence-based care practices, WHO published a list of Standards for improving the quality of maternal and newborn care [3] and other guidelines, such as the intrapartum care recommendations for a positive childbirth experience [4]. These documents empathize with the concept of women-centered respectful maternity care (RMC), and the White Ribbon Alliance’s rights-based outline of an RMC charter [5].

Even before the COVID-19 pandemic, research on obstetric violence [6], on disrespect and abuse [7], or on mistreatment during childbirth [1] showed that neglective care, restrictions in companionship of choice, or unjustified limitations of bodily autonomy have detrimental consequences on the health and wellbeing of women who give birth. The pandemic has exacerbated those issues: measures to contain the spread of the virus and high rates of infection have had an impact on the provision of care, potentially restricting women’s rights and increasing the risk of experiencing different forms of mistreatment or violence while giving birth [8].

In those conditions, it is even more challenging to preserve a woman’s birth integrity. With the idea of birth integrity, we postulate that every birthing person’s autonomy, self-determination, wholeness, and human rights need to a priori be respected and protected to preserve their integrity. Therefore, we aim to capture how women perceived giving birth as an embodied experience – and whether their integrity was violated or preserved throughout [9, 10]. An embodied perspective upon birthing includes the feelings, emotions, physical sensations, the social surrounding of birthing, and the broader societal contexts [11, 12].

To capture birth integrity, the analysis of maternal voices and expressions outside of (or at least: besides) traditional research settings is essential and offers insights into what matters to women. There is a need to value this experiential knowledge as scientific [13] and take more perspectives on health than classic biomedical knowledge production does, thereby adopting a feminist approach to women’s health research [14]. One growing source of such knowledge is social media, which is now used by the vast majority of the population in most contexts. For example, in Germany, where this study is set, 90% of female internet users aged 20–29 and 85% aged 30–39 were social media users in 2020/21 [15]. The preferred medium for pandemic-related information among adults over 30 years old was Facebook [16]. With such a reach, social media can be a valuable alternative source of data and evidence, especially in times of disruptive changes similar to the COVID-19 pandemic [17]. On emerging issues, social media data can indeed provide a snapshot of important societal concerns worth investigating further in future research [17]. More generally, social media data is used in research for content analysis, surveillance, engagement, network analysis, recruitment, and intervention [18, 19].

During pregnancy and motherhood, in particular, social media play a non-negligible role, as women use them to seek out information as well as emotional and social support. Online groups and pages can provide them with a safe place to discuss birth-related questions and intimate topics [20, 21]. Among the stages of pregnancy and birth, labour is one of the most worrying to women due to the uncertainty and lack of control surrounding the event [22]. Women actively seek information on birth, which was the main topic in a largely automated analysis of 262,238 posts in online birth club forums [22]. Online, women also seek reassurance, social support [23–25], and empathy from peers [26, 27]. The sharing of experiential knowledge is a crucial component of the pregnancy and birth focused platform and forums [26].

Our objective was to understand better the experiences of women giving birth in the context of the COVID-19 pandemic in Germany. To do so, we explored themes discussed by mothers on social media, and through the lens of birth integrity, paying attention to what may help protect birth integrity and, similarly, what has the potential to violate a birthing person’s integrity.

## Methods

### Data collection

We analysed comments posted on a Facebook public page as a reaction to the dissemination campaign of a survey on experiences of maternity care during the COVID-19 pandemic. Indeed, to acquire participants for a study, bloggers and social media parenting pages were asked to disseminate study information on different platforms (i.e. social media platforms as well as personal blogs and websites). The data of this paper is a by-product of this dissemination strategy, composed of comments generated by a single post promoting the research study. The post was hosted by the Facebook page of a well-known motherhood and parenting blog. This blog is not a birth-focussed blog, nor an advocacy blog. The post was short and neutral: it read, “Looking for participants” and shared the link to said study. Users then reacted to this post, either by using a “like” button to show interest, or by commenting under the post.

### Data analysis

The data comprised three main components suited for analysis: the written comments, the emojis inserted in the comments and aimed at expressing a range of emotions [28], and the reactions to the comments, which can signify with yet another emoji or through a more neutral reaction button a supportive position, empathy or opposition. A last type of information of importance was the contextualising elements of the birth (e.g., type of facility, type of delivery, month of delivery) that were either nested in the written comments or retrieved from the time marks of the comments. In relation to our topic of interest and an ever-evolving pandemic situation and accompanying measures, these elements were also of relevance.

We took screen-shots of the comments and then copy-pasted them in an Excel document for analysis. We followed the steps of thematic content analysis [29] as described by Nowell et al., 2017 [30]. AL, CM, and SB-Z all read the data thoroughly, several times. AL then started the inductive coding, which was regularly discussed with SB-Z and CM. Once the list of codes seemed finalised, AL began the axial coding to create categories of codes, or “topics”. CM and SB-Z then analysed the topics through a birth integrity lens to arrive at the emergence of themes or shared patterns of meanings. For the interpretation of the emojis, we referred to the emoji dictionary Emojipedia [31].

AL had no experience of giving birth, SB-Z and CM did. SB-Z and CM discussed with AL collaboratively and challenged the characterisation of codes based on their understanding of pregnancy and birth. In identifying and discussing the attribution and organisation of codes, all authors sought to be aware of how their positions may have impacted their interpretation of the data [30, 32].

Following analysis, quotes were selected based on their representativeness for the findings and were subsequently translated from their original language (German) into English.

### Ethical considerations

Questions of consent and privacy are key points of research using social media data [33]. There is no clear consensus on what makes posts public or private and, therefore, exploitable for research purposes. There are, however, recommendations to think carefully about ethics. Here we used posts written on a public page (not in a private page or group), whose authors are expected to assume that their contributions are visible to all, and in the public domain [34]. However, we are aware that their intention when posting was to share experiences with peers and not necessarily become research participants [35]. Since fetching consent from all the participants felt unrealistic (too many, likely low response rate) and unethical (invasion of privacy, harassment, giving the impression they are being observed) [34], we chose to treat the comments as secondary data and took a series of steps that would prevent identification of the contributors: : (i) we removed the identity of the contributors (each contributor or “Author” was assigned a number, e.g. A1); (ii) we removed any contextual information (specific location, day of birth) that could allow identifying contributors; (iii) we translated the quotes from German into English; (iv) we checked that quotes entered into Google (in English and back-translated into German) did not retrieve the original comment, also when searching for the platform in which it was posted, and within the platform search tool; (v) we waited more than six months after the publication of the post to start sharing the results.

Electronic correspondence between the corresponding author and the data protection office of Bielefeld University confirmed the viability of the study: since all the data were publicly available and considered secondary data, we were advised that the project did not require ethical review.

## Results

**Fig. 1:**
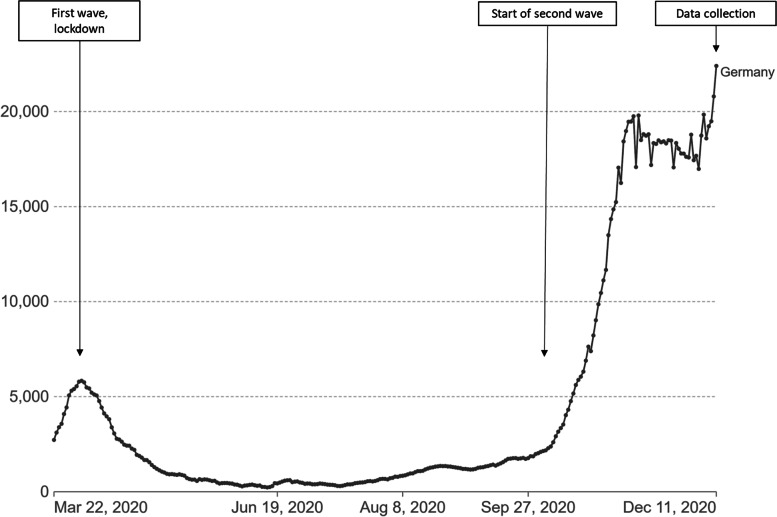
Daily new confirmed COVID-19 cases (7-day rolling average) and study timeline. Source: Figure extracted from “Our World in Data” website, based on Johns Hopkins University CSSE COVID-19 data, adapted by the authors

The dissemination post went online at the end of the year 2020 (exact date retracted to prevent identification). At the date of data download, a week later, it had been liked 86 times, shared 48 times, and commented 213 times (direct comments to the post and replies to comments). Each comment also generated its own share of engagement or reactions in the form of “like” buttons and emojis. Many of the contributions were tags to point out the study to women who met the requirements or might be interested in participating (*n* = 68). Another set (*n* = 17) was signifying own participation (e.g. “participated!”). One comment was someone using the post as an opportunity to advertise a product. This left 127 written contributions from 69 individual contributors for the content analysis, as well as contributions from further users in the form of reactions, i.e. “likes” and emojis. The majority of comments generated 1 to 5 reactions, 5 comments more than 10 reactions, and one comment 109 reactions. The most mentioned topics and related themes created through the analysis pertain to (i) own mask-wearing, (ii) the presence or absence of a birth partner and supportive care at different stages of the birth process, and (iii) visiting rules, i.e. the (im)possibility to welcome visitors in addition to the birth partner. A fourth, less explicit theme runs through all topics and relates to the way COVID-19 measures are understood and accepted or rejected. Not all women indicated when they had given birth, but for those who did, we mention the birth month after their identifier. For a better understanding of the epidemiological context, Fig. 1 plots the study timeline against the COVID-19 incidence in Germany between March 2020 and December 2020.

### Own mask-wearing as a physical and psychological threat to birth integrity

The comments on own mask-wearing during birth refer to the different phases of labour, birth, and the postpartum stay at the maternity ward. Overall, the comments indicate that mask-wearing in communal spaces (outside the patient room, e.g., corridors) and when healthcare professionals entered the patients’ room was obligatory for almost all women. One woman reported having to wear a mask “day and night”, as a healthcare professional could enter the room any time (A126, April).

Some women were not obliged to wear a mask during birth, including women with a planned caesarean section (A28, A106), and others having a vaginal birth (e.g. A120):

Fortunately, I didn’t have to wear one [mask] either. The midwife immediately said that the masks can be removed, you cannot give birth with it (A120).

Still, many comments disclose that women were requested to wear a mask during the active phase of labour and until either a healthcare professional or themselves got rid of the mask:

Mask on, even during the birth. But when it went into full labour, the thing flew through the delivery room once. Nobody said anything. (A96, September)

I always wore a mask in the room when someone came in. […] in the delivery room during CTG [Cardiotocography] and other exams [I was] always wearing a mask. During birth, when things got really serious, I was fortunately allowed to take the mask off. (A55, November)

In the beginning the mask had to be on, during birth, it didn’t matter at some point, only the midwife and the doctor had theirs on. We had to put ours back on when we went to the ward. (A84, September)

The comments demonstrate a discrepancy between the formal instruction to wear a mask and the actual, more flexible handling from the healthcare professionals when women went into full labour.

What the above quotations have already indicated (omission of the mask as a relief), is explicitly expressed in the following comments, demonstrating that mask-wearing at any time during birth (including during expulsion) was perceived as an unpleasant and stressful experience:

I also had to wear a mask. Quite horrible. (A22)

Oh, I’ll take a look at that [survey]. If it helps that women do not have to give birth with a mask, like me, it would be a success. (A111)

This last comment by A111 was met with 16 supportive reactions, primarily emojis expressing sadness and shock. It also led to a controversial discussion as one contributor claimed the salutary role of masks in protecting the healthcare professionals from infection in a rather offensive tone, e.g., claiming that women make “a huge drama out of everything and get hysterically worked up about it” (A26). Most of the subsequent commentators solidarised with the women having had negative mask-wearing experience and gave reasons against wearing a mask during active labour, e.g., according to their midwife, “oxygen supply is the most important thing”, why mask-wearing is not requested in their hospital (A23) or pointing out that the German midwifery association clearly states “no to mask-wearing” (A30). One contributor held accountable the hospital management and policy for providing midwives with “appropriate masks (FFP2) for their own protection” and:

(…) let her [the parturient] damn breathe freely. Not because otherwise, she will suffocate (…). But because she is about to give life to a little human being and breathing is essential. (A24)

For many who mentioned mask-wearing without emphasis and without diving deeper into the pros and cons of general mask-wearing, we can assume that they share a general understanding and acceptance of mask-wearing as a measure to contain the spread of SARS-COV-2. However, the discussion specifically about mask-wearing during the active phase of labour reveals that acceptance of the rules comes with limits and that women explicitly articulate the difference between what seems an acceptable public health measure and what is considered an infringement to the physiological process of birth or to their bodily integrity.

### Limited presence of a companion of choice during birth and lack of supportive care: women at risk of abandon

Whether a companion of choice was allowed to accompany the women throughout labour and birth varied: while some women who had undergone a planned caesarean section had their partners with them throughout the procedure (A106, A28), one woman had to deliver without her partner:

Husband was not allowed to attend the caesarean section! (was worst part of the [experience] for me) (A79, May).

Many women shared the experience of having to manage the contractions alone and that their partner was only allowed to join when they went into active labour (A82, August; A19, September; A84, September) or, when after going into labour, a caesarean section was performed:

My husband was only there for the C-section. During labour, I was alone - and the 3 days afterwards on the ward [I was] also alone [sad emoji and crying emoji] (A76, March).

My husband was not allowed in the delivery room until it started. (…) I could only call him after the water broke. He almost missed the birth [crying emoji] (A96, September).

This woman, whose birth was induced, was alone until the onset of labour as well. Nevertheless, she felt well supported “since everyone at the hospital was really totally sweet”, and her husband was called in time to attend the birth (A124, November). Her positive perception of being well cared for seemed to balance out the absence of her partner, at least partially.

In contrast, A122 not only had to cope with labour and birth on her own but in addition was confronted by a lack of supportive care that added to her overall negative birth experience,

I gave birth (…) in April alone, without my husband. Even though it was the third child, it was just awful because the support was lacking. Also, the staff was hardly there, so I was alone in the delivery room for 11 h and had to go through labour alone. It wasn’t until the final spurt that a super lovely midwife came and supported me (…) this [experience] was awful for me, and I struggled with it for a long time (A122, April).

Another woman who also gave birth "alone" in April was similarly denied supportive care:

[I gave birth] in April, delivered alone, and no visit of my husband or child was allowed afterwards.[…] [I] was also not allowed to be present at any examination of the child. (A126)

Her comment generated 109 reactions, with many emojis manifesting the anger, shock, sadness and empathy of the other contributors. The complete isolation of the woman, including from her newborn during examinations, was upsetting to many, with the words “horrible” and “inhuman” being used to describe the situation.

The absence of a birth partner and supportive care was generally seen as detrimental to the birth experience and considered as an extreme (somewhat unjustifiable) measure that was going “too far”. The women who were allowed to be accompanied throughout their hospital stay showed gratitude and acknowledged how “lucky” they had been compared to those who were denied such support.

### Limited visiting hours and restricted access: contrasting impacts on birth integrity

The comments reveal restricted partner visiting regulations for all patients, ranging from a complete ban on visitors, including partners presence during and after birth (A122), to restricted visiting hours (A19):

My husband wasn’t allowed to visit us, so he didn’t see his little nugget live until he picked us up from the hospital two days after delivery. (A122)

After the birth, there were visiting hours of 3 h for my husband. He also had to go home two hours after the birth and was allowed to come back at visiting hours… On the day of discharge, he was also not allowed to pick us up from the room, there haven’t been visiting hours… (A19, September).

Welcoming other visitors, including the newborn’s siblings, was not possible. Due to those restrictions and sometimes lack of childcare for older siblings, some partners had to refrain from visiting (A84):

My husband could have visited from 2 to 8 p.m., but he didn’t because my son was not allowed to come to the hospital. Other visitors were not allowed to come. (A84, September)

My husband was there [hospital] once for exactly one hour because he was not allowed to bring our three year old, and we had no one to watch her. I discharged myself on the 3rd day after having many discussions. (A119, June)

The clinic had already sealed itself off. Visits for an hour a day and without children. On the third day, I was discharged at my own request. (A42, March).

Those rules negatively affected many women, which sometimes led them to request a shorter length of stay and early discharge from the hospital (as seen in the two last comments above)^2^. In some cases, women decided to circumvent the rules by choosing to deliver in settings that would allow them more freedom, for example, a smaller hospital with “loser rules” (A26, A28) or a home birth (A109):

We didn’t want all these rules. We gave birth at home. Midwife no mask, me none and my husband none. Visits from the first hour the way I wanted it. Thank you, COVID-19 for this indescribable and self-determined birth. (A109)

Although the limitations of partner’s visits and the ban for siblings of the newborn was for many women problematic, the limited visiting hours and low number of visitors in the hospital was the most appreciated pandemic-related measure.

Much quieter without other visitors compared to my 1st [time at the maternity ward]. (A10)

It was quiet and relaxed in the ward. Lovely. No crowds of visitors. (A96, September)

Other women shared these feelings and experiences, commenting how the maternity ward felt calm and relaxing, thanks to the absence of visitors (A58, A96).

New mothers and healthcare professionals alike saw the relative emptiness and quiet of the maternity wards as an opportunity to rest, heal and recover. It also provided a protective setting for the important developments in the postpartum phase (e.g. breastfeeding) and the nurturing of a bond between the mother and her newborn:

My husband was allowed to come daily at visiting time, but other visitors were not, which did not bother me at all. We were able to be in peace and quiet, and the staff on the ward also told us that the women recover so much faster from birth, the milk comes in more quickly (A58, October).

It was wonderfully quiet in the hospital, and we could relax without the stress of visitors, and I could recover. (A38, April)

Similar to own mask-wearing, the restrictions put on the number of visitors and visiting hours were, to some extent, understood and accepted by most women. Several contributors even identified in quieter surroundings positive aspects of the pandemic and an opportunity to revel in the postpartum period. But common to all testimonies remain the need to take into account, as much as possible, the personal circumstances, wishes, and expectations of every woman, e.g. the one with other children, the one who prefers to be discharged early, the one who needs quiet.

## Discussion

Our findings emphasise deficiencies in maternity care as experienced by birthing women during the pandemic in Germany and how those deficiencies are a threat to the parturients’ birth integrity during labour or birth. In the comments, the most mentioned topics were own mask-wearing, having a companion of choice during birth and supportive care, and restriction on visitors. Those topics also generated the most reactions, revealing compassion from other women and mixed feelings about health measures, from acceptance to shock and anger.

Especially at the beginning of the pandemic, some of the measures and changes in the organisation and delivery of maternity care have threatened rights to respectful maternity care. This led civil societies globally and international organizations (e.g., World Health Organization, International Confederation of Midwives) to call for less restrictive measures [36–38] with a particular emphasis on allowing birth partners, avoiding unnecessary interventions, and guaranteeing opportunity for postpartum bonding between the newborn and their mother. Such infringements of basic birth rights are visible in our data, as illustrated by the experiences of several women, who shared that they had given birth “alone”, which was perceived as traumatising and impacted their wellbeing negatively. Going into labour and birth already makes the parturient vulnerable. Having to face this unpredictable situation on one’s own devices and against one’s will without the presence of a close person is a threat to birth integrity. The common absence of a companion of choice during birth is particularly concerning, as research indicates that a companion of choice provides practical, emotional and informational support and helps women having a positive birth experience [39]. The presence of birth companions has been shown to positively impact the physiological progress of labour, including decreased use of birth interventions [40]. The WHO also recommends that women should be allowed to have a companion of choice being present during birth regardless of whether or not they are infected by COVID-19 [37]. Current measures (still ongoing in 2022) restricting the access of partners and fathers to maternity wards threatens the slow progress made in the past few years regarding the involvement of fathers in pregnancy and maternity care [41]. Beyond the direct harm that they may cause during the birth process, they relegate partners and fathers to the role of simple visitors, stripping them from their role as valued actors of the birth [42]. Concerning the provision of respectful maternity care, our data shows that an empathic and supportive birth accompaniment by a healthcare professional has the potential to make up for worsened conditions, especially when giving birth without a companion of choice. These findings are supported by studies on the positive effects of continuous care support [43].

Wearing a mask during delivery is a preventive measure to reduce the risk of infection of both the parturient and healthcare professionals [44]. This strategy has also been performed in past epidemics when women confirmed or suspected H1N1 positive wore surgical masks during labour and delivery [45]. Since the start of the COVID-19 pandemic, mask-wearing has been one of the first and most consistent measures implemented in maternity wards, albeit with variations across facilities. According to a study conducted in Japan at the beginning of the pandemic, women were requested to wear a mask during labour and delivery in 64,6% of the facilities [44]. Several associations in Germany (e.g., the German midwifery association, the German Society for Obstetrics and Gynaecology) stated that mask-wearing during active labour is to be discussed per individual case and should be enforced only if the COVID-19 status of the parturient is unknown or positive [46, 47]. The midwifery association emphasised that healthcare professionals are supposed to wear protective gear in first place when being confronted with a COVID-19 positive parturient and that a “woman should wear a facial mask if and as long as she tolerates it” [46]. As the most spoken about topic in our dataset, mask-wearing has been a genuine concern for women giving birth during the pandemic and comes with the potential to violate birth integrity, as suggested by testimonies of having felt “horrible” and of relief when masks were no longer worn. This resonates with research on maternity care during the pandemic [48] and with reports in the media on how mask-wearing during labour and birth can cause panic, fear, and claustrophobia [49–51]. There is, however, no study to date which specifically investigates the impact that it can have on birth experience and postpartum health. The sharing of experience via (social) media highlights the urgency for epidemiological research and health policy to take seriously what affects maternal birth experiences and potentially violates birth integrity.

Another important finding of our study concerns the visiting rules in maternity wards. Despite the fact that some mothers were saddened because their older children were not allowed to visit (and their partners only to a limited extent), many comments reveal that restrictions on visitors had positive effects on the mothers. Many claimed feeling more relaxed, enjoying the quietness of the maternity wards, and having undisturbed time for bonding and breastfeeding. Similar findings emerged from interviews with mothers and healthcare professionals in 2021 in Germany [42] and from a global review of maternity care experiences in the early stages of the pandemic [8]. Considering the potential of such circumstances on health and wellbeing, we think it is crucial to research further the conditions that protect new mothers in the early postpartum stage and enable a safe space in a period of hormonal, emotional and physical transitions. Protecting birth integrity includes an understanding that the early postpartum stage entails vulnerability as well. Sheltering this vulnerability could involve allowing exclusive time for the new parents, a reassessment of the standard length of hospital stay, protected space and time from unwanted visitors, and the systematic availability of support measures for breastfeeding.

Communication about the pandemic-related measures is essential. Our data show that a good understanding of the motivation behind the measures (e.g., protecting healthcare personnel) can lead to a better acceptation and fewer negative experiences. It points towards preparedness and the importance of information and communication in healthcare settings [52]. Insecurities related to catastrophic events can affect maternal and infant health and result in preterm birth, low birth-weight and maternal mood disorders [53]. An obvious solution to mitigate this risk is to explain and prepare, and more generally, to communicate in a respectful and adapted manner.

With women voicing their concerns and reporting their experiences, social media allow for the emergence of under-explored topics and emphasises what matters to women during and after birth [17]. Similar to Wexler et al., 2020, our findings are distinct from previous work that directly queries women instead of looking at their internet usage [22]. Our findings on own-mask wearing, for example, have so far not been central to research on maternity care [8]. As emphasised in a qualitative interview study, the pandemic response is not only about keeping women physically safe but also managing the emotional toll brought about by the changes in maternity care [54]. It is part of upholding feminist values in health research to consider multiple perspectives on the health of populations and include testimonies that fall beyond the scope of traditional data collection [55]. Our study is an example where social media can be used to capture important concerns and define research questions while listening to women’s voices [56]. The findings could inform the future developments of the current pandemic and similar events, as well as a more general debate about optimal conditions for respectful maternity care. Acknowledging the existence of traumatising birth experiences calls for strategies that support women and mitigate the potential negative effects of such experiences on maternal mental health [57], bonding and parenting [58], and future use of healthcare services [59].

### Strength and limitations

Since our results are based on comments under a social media post about giving birth during the COVID-19 pandemic, respondents expressed themselves freely and without being guided by researchers [48]. The comments provided new insights into how mothers are affected by giving birth during the pandemic in Germany –a country for which evidence on experiences of maternity care during the pandemic has been scarce so far. Based on our results, fears, concerns, and experiences were identified and could inform future practice. However, this study has limitations. First, a selection bias may have operated so that mothers who experienced more restrictions due to the pandemic were more likely to share their experiences. Secondly, there were limitations due to the data collection itself, as we obviously only captured the experiences of women who use Facebook. Our findings are not representative of all women giving birth during the pandemic and we do not have socio-demographic information on the contributors. Still, based on observation of the hosting page content, we can assume that the main public of this page is composed of white middle-class women. Third, the data only represent experiences made in the German context. Last, we analysed only few comments (*n* = 127), compared to several thousands in other studies such as Gui et al., 2017 [26] for example. However, we believe that the strength of our dataset is that all contributions are answers to a single post and therefore focus on the same specific topic, i.e., experiences of birth within a limited time-period of the pandemic.

## Conclusion

By analysing social media comments through the concept of birth integrity, we highlight how own mask-wearing, supportive care, the presence of a birth companion of choice, and visiting rules shape the experiences of birth during the pandemic. Exceptional pandemic circumstances have introduced new parameters in maternity care, some of which appear acceptable, necessary, or beneficial to women, and some of which constitute real threat to birth integrity. Our research calls for the investigation of the long-term impact of those violations of birth integrity and the reassessment of the optimal conditions of the delivery of respectful maternity during the pandemic and beyond.

## Data Availability

The datasets used in the current study are available from the corresponding author on reasonable request.

## Footnotes

1 We acknowledge that persons other than cisgender women also give birth and we value their experiences. In this article, we understand the word “women” as an inclusive term.

2 To give context to those requests: in Germany, it is customary for the parturient to stay a whole week in the hospital following the birth of her baby. This stay, also known as “Wochenbett” (week-long bed) is a protected time supposed to allow her to fully recover from the birth and to bond with her child.
